# Special Issue: Advanced Research in Pediatric Radiology and Nuclear Medicine

**DOI:** 10.3390/children10121917

**Published:** 2023-12-12

**Authors:** Curtise K. C. Ng

**Affiliations:** 1 Curtin Medical School, Curtin University, GPO Box U1987, Perth, WA 6845, Australia; curtise.ng@curtin.edu.au or curtise_ng@yahoo.com.hk; Tel.: +61-8-9266-7314; Fax: +61-8-9266-2377; 2 Curtin Health Innovation Research Institute (CHIRI), Faculty of Health Sciences, Curtin University, GPO Box U1987, Perth, WA 6845, Australia

The importance of pediatric radiology and nuclear medicine is increasing. There is currently more demand for this subspeciality of radiology compared to the past [1]. Research in pediatric radiology and nuclear medicine is essential for continuous growth of this subspeciality and evidence-based practice to improve pediatric patient outcomes. However, it is noted that more support needs to be provided to pediatric radiology and nuclear medicine researchers to share their research findings [2]. The journal *Children* is forward-looking and initiated the ‘Advanced Research in Pediatric Radiology and Nuclear Medicine’ Special Issue to provide a platform to facilitate the rapid sharing of research findings and promote evidence-based practice in 2022. It has been my honor to be the Guest Editor of this Special Issue and its second volume over the last two years. So far, 13 articles have been published in volumes I (https://www.mdpi.com/journal/children/special_issues/Pediatric_Radiology_Nuclear_Medicine, accessed on 28 November 2023) and II (https://www.mdpi.com/journal/children/special_issues/0ZZ2T5PNBY, accessed on 28 November 2023) of this Special Issue and I would like to thank all authors for their valuable contributions.

Collectively, the 13 contributions cover all common medical imaging modalities in pediatric radiology (plain radiography, ultrasound, magnetic resonance imaging (MRI), computed tomography (CT), fluoroscopy and interventional radiology (IR)) and nuclear medicine (single-photon emission computed tomography (SPECT) and positron emission tomography (PET)). Eight contributions specifically focus on plain radiography (contributions 1 and 2), ultrasound (contributions 3–6) and MRI (contributions 7 and 8). This pattern appears in line with the current trend of pediatric radiology that plain radiography is still the most common radiological examination type, but there are increasing uses of ultrasound and MRI to replace CT due to its high radiation dose, which is a serious issue for children who are more vulnerable to the potential harmful effects of ionizing radiation [3,4]. Hence, contributions 9 and 10 systematically review the radiological examination dose issue in children with congenital heart disease (CHD) and use of artificial intelligence (AI) for dose reduction, respectively. Nonetheless, pediatric radiologists seem more interested in AI for addressing their workload burden [1] because burnout in pediatric radiology is an increasing problem, as per the recent literature [5,6,7]. Contribution 11 reveals that the current AI technology is able to support less experienced pediatric radiologists in image interpretation, but further research is needed for its wide adoption. Contribution 12 further explores the potentials of AI (specifically generative AI) for pediatric radiology and nuclear medicine, and reports that generative AI can be used for pediatric disease diagnosis and image data augmentation, quality assessment, reconstruction, segmentation, synthesis and translation. Nowadays, medical images are viewed on computer monitors as a standard practice [8]. However, contribution 13 illustrates an extended use of medical images for better visualization of pediatric CHD through three-dimensionally printed models, which provide added value for the diagnosis and treatment of this disease.

Although the current coverage of volumes I and II of the ‘Advanced Research in Pediatric Radiology and Nuclear Medicine’ Special Issue seems comprehensive, according to the research trend of pediatric radiology and nuclear medicine, many pediatric pathologies, imaging techniques and radiology education issues have still not been addressed in this Special Issue [2]. I would like to encourage pediatric radiology and nuclear medicine researchers to consider making further contributions to this second volume (https://www.mdpi.com/journal/children/special_issues/0ZZ2T5PNBY, accessed on 28 November 2023) in 2024. In this way, we can ensure continuous growth of this subspeciality and evidence-based practice to improve pediatric patient outcomes.
